# An Unusual Case of Aortic Stenosis in Systemic Sclerosis

**DOI:** 10.7759/cureus.1303

**Published:** 2017-06-01

**Authors:** Mustafa Kagalwalla, Medhavi Gupta, Umme Haani Malwi, Nazia Hussain

**Affiliations:** 1 Internal Medicine, Mount Sinai Saint Luke's Hospital Center; 2 Medical Student, Gandhi Medical College, Bhopal, MP; 3 Rheumatology, Mount Sinai Saint Luke's Hospital Center

**Keywords:** systemic sclerosis, aortic stenosis

## Abstract

Systemic sclerosis is a connective tissue disorder that frequently involves the heart. All cardiac structures can be involved but aortic valve involvement is rare. We report a case of an 83-year-old man with a history of systemic sclerosis presenting with shortness of breath. A transthoracic echocardiogram revealed severe aortic stenosis that could not be explained by other causes. These patients often pose challenges in management as they are not surgical candidates. Transaortic valvular implantation is a viable option for these patients.

## Introduction

Systemic sclerosis is a connective tissue disorder with multisystem involvement. It is characterized by diffuse fibrosis of the skin and internal organs. Systemic sclerosis has a strong female predominance (4.6:1) and the peak age of onset is 30–50 years [1]. The causes of systemic sclerosis are not clear, but autoimmunity, endothelial cell damage, and increased production of extracellular matrix likely play key roles [2]. The pathologic hallmark of the disease is the combination of widespread capillary loss and obliterative microangiopathy, together with fibrosis. Systemic sclerosis commonly involves the skin, lungs, gastrointestinal tract, heart, and kidneys. In the heart, systemic sclerosis can involve the endocardium, myocardium or pericardium. Conduction system fibrosis is also commonly seen [1]. Myocardial involvement is common, and with sensitive tools, it has been estimated to occur in up to 100% of the patients [3]. Clinical evidence of myocardial disease may be seen in 20% to 25% of the patients [4]. When cardiac involvement appears clinically evident, it is recognized as a poor prognostic factor [5].

## Case presentation

**Figure 1:**
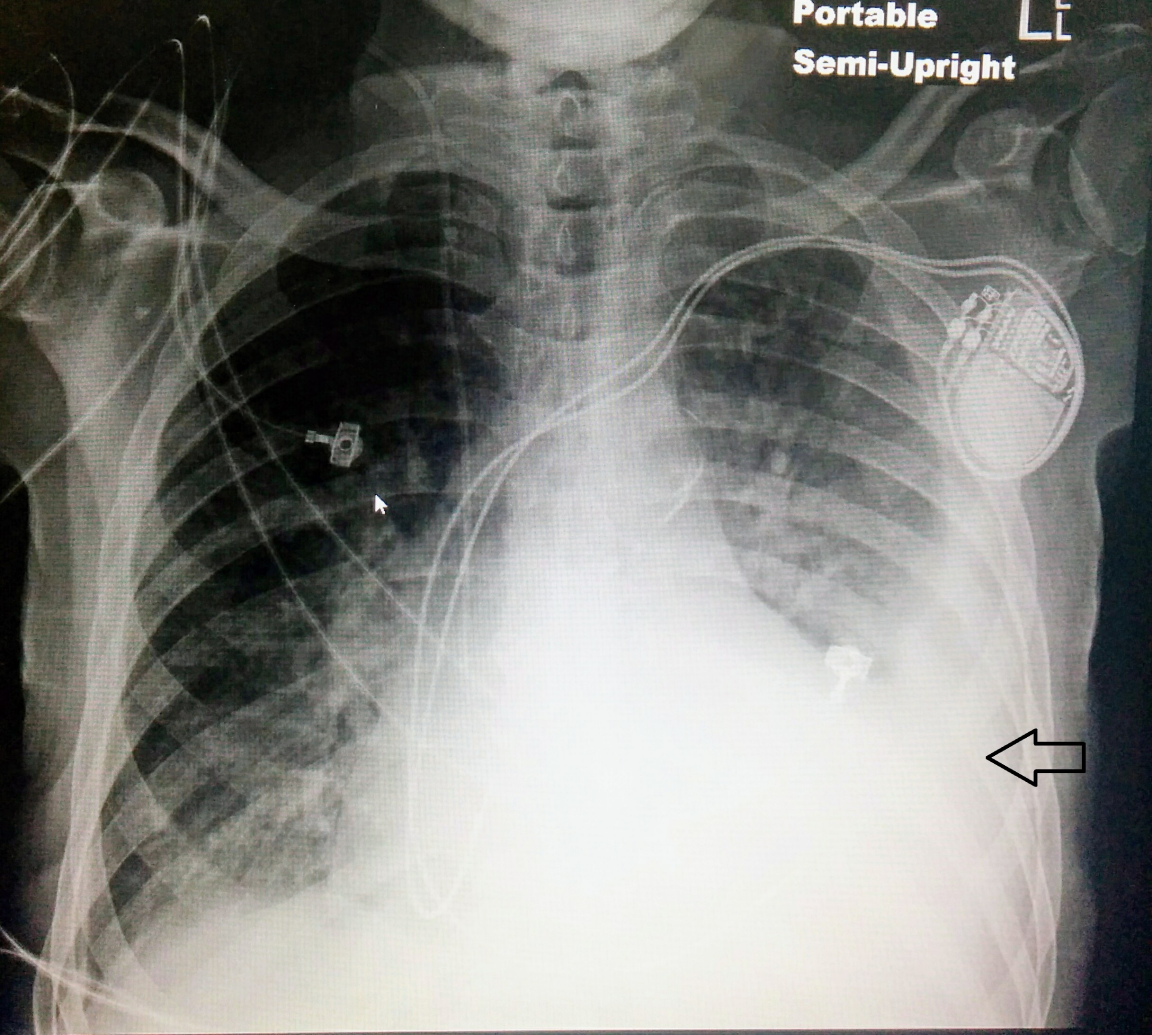
Portable chest X-ray (AP view) showing left sided pleural effusion (arrow) and vascular congestion AP - anteroposterior.

**Figure 2:**
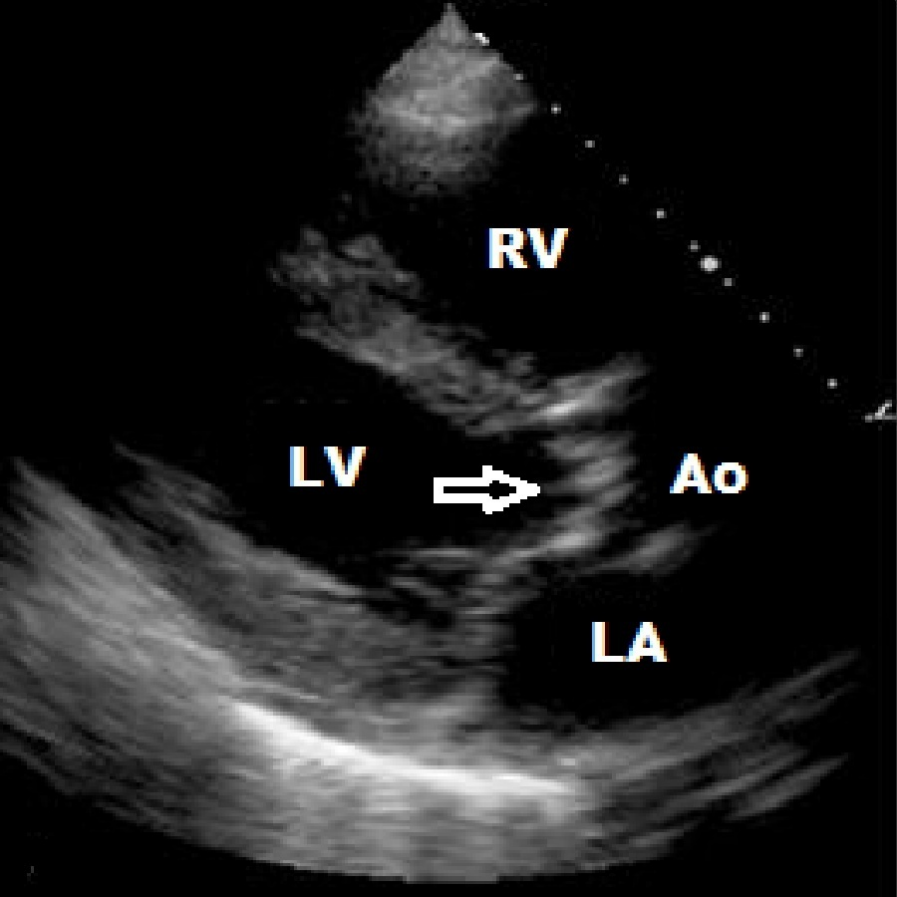
Transthoracic echocardiogram (parasternal long axis view) showing severe aortic stenosis (arrow) RV - right ventricle, LV - left ventricle, Ao - aorta, LA - left atrium.

An 83-year-old man with a history of systemic sclerosis with diffuse cutaneous involvement and complicated by infiltrative cardiomyopathy, heart failure with preserved ejection fraction and complete heart block (with a permanent pacemaker) presented with progressive exertional shortness of breath for two months. The vital signs at presentation were as follows: temperature 98.9 degrees Fahrenheit, respiratory rate (RR) 24/min, heart rate (HR) 80/min, and blood pressure (BP) 112/78 mmHg, saturating 90% on room air. The physical exam was remarkable for bibasilar crackles, a systolic murmur at the left lower sternal border, bilateral lower extremity pitting edema, and a diffusely thickened skin. An electrocardiogram revealed an atrial sensed, ventricular paced rhythm with appropriate discordance. The laboratory work was significant for a creatinine of 1.5, troponin of 0.11 (which subsequently trended down to 0.06), and b-type natriuretic peptide (BNP) of 2600. A chest X-ray revealed a left-sided pleural effusion with vascular congestion (Figure 1). A transthoracic echocardiogram revealed an ejection fraction of 35%, a tri-leaflet aortic valve with the calculated aortic valve area of 0.8 cm^2^, a peak transaortic velocity of 4 m/s, and a mean gradient of 35 mmHg consistent with severe aortic stenosis along with moderate pulmonary hypertension (Figure 2). Coronary angiography revealed non-obstructive coronary artery disease. Right heart catheterization showed a mean aortic valve gradient of 30 mmHg, a calculated aortic valve area of 1 cm^2^ and a left ventricular end-diastolic pressure of 16 mmHg. The patient was managed with aggressive diuresis and given oxygen support. The symptoms were attributed to severe aortic stenosis. The patient underwent aortic valve valvuloplasty, which did not improve the symptoms. The patient was deemed unfit for surgical valve replacement due to being a high risk case from his comorbidities and underwent transaortic valvular implantation with an improvement in symptoms.

## Discussion

Systemic sclerosis is an autoimmune disorder of the connective tissue involving fibrosis of the skin and internal organs. Even though the involvement of the heart is very common, valvular involvement in systemic sclerosis is uncommon and most often involves the mitral valve causing mitral valve prolapse [6]. Aortic stenosis is an extremely rare complication [7]. In 2007, De Groote, et al. evaluated 570 patients with systemic sclerosis for cardiac abnormalities with Doppler echocardiography and found aortic stenosis in only 3.3% of the patients [8]. The etiopathogenesis of aortic stenosis in systemic sclerosis might include underlying inflammatory burden leading to endothelial dysfunction, enhanced activity of fibroblasts accelerating the processes of aging and hemodynamic stresses [9].

Management of patients with severe aortic stenosis is often very challenging. These patients have multiple comorbidities including interstitial lung disease, pulmonary hypertension, ventricular dysfunction, and arrhythmias. These comorbidities make these patients extremely high risk cases for surgical interventions. Furthermore, the intraoperative period requires meticulous management as these patients tend to have difficult intubation with increased risk of regurgitation due to reduced esophageal motility and increased risk of bleeding due to telangiectasia. Other concerns include induction of Raynaud’s phenomenon to internal organs due to hypothermia during the surgery and immunosuppression leading to increased infectious risk [10]. Hence, aortic valve replacement surgery is often not available to these high-risk patients. Transaortic valvular implantation (TAVI) helps avoid general anesthesia and intubation. Also, it is associated with less complex peri-procedural management and shorter in-hospital stay. TAVI could be a practical therapeutic option for inoperable aortic stenosis (AS) patients. Our patient successfully underwent TAVI and his symptoms are much improved now.

## Conclusions

Aortic stenosis is a rare complication from systemic sclerosis. Its management poses many challenges as aortic valve replacement is not an option for these high-risk patients. Transaortic valve implantation is a viable option for these patients.
